# Research progress on biomarkers associated with neonatal respiratory distress syndrome

**DOI:** 10.3389/fped.2026.1811632

**Published:** 2026-05-18

**Authors:** MaoTing Xiong, JiaHong Deng, Jing Zhao

**Affiliations:** Department of Neonatology, Affiliated Hospital of North Sichuan Medical College, Nanchong, China

**Keywords:** biomarkers, lung ultrasound score, neonatal respiratory distress syndrome, preterm infants, pulmonary surfactant

## Abstract

Neonatal respiratory distress syndrome (NRDS), resulting from pulmonary surfactant deficiency, continues to be a significant cause of morbidity and mortality in preterm infants. Conventional diagnostic and therapeutic approaches have notable limitations in early warning and prognostic stratification. Consequently, biomarker research has emerged as a promising new direction in this field. We systematically searched PubMed, Web of Science, and Embase for studies published from January 2010 to December 2025, focusing on those that evaluated the application of inflammatory markers, microRNAs, protein and peptide biomarkers, and lung ultrasound score (LUS) in NRDS. The main findings were as follows: ① inflammatory biomarkers: IL-6, IL-17, and related markers were positively associated with disease severity, although their specificity remained limited; IL-37 showed potential as an anti-inflammatory therapeutic target. ② microRNAs: miR-375, miR-363, and others show better performance when combined with lung ultrasound and blood gas parameters, but this is based on preliminary single-center data. ③ Protein biomarkers: ANGPTL4 has diagnostic and prognostic value; combining it with lung ultrasound increases AUC, but evidence is still early and lacks external validation. ④ Clinical tools: Lung ultrasound, as a non-invasive real-time bedside tool, has good diagnostic performance and is convenient for repeated exams, but its combined use with emerging molecular markers requires further study. ⑤ Machine learning: although it has shown advantages in multimodal data integration, most models still lack external validation, and concerns regarding overfitting and limited interpretability continue to restrict clinical implementation. Overall, combined biomarker strategies and tools like lung ultrasound have shown promise in some studies, but current evidence is insufficient for broader clinical use. Most emerging biomarkers, including miRNAs and peptide markers, face challenges with assay standardization, independent validation, and prospective evaluation. Future studies should aim to translate biomarkers from laboratory discoveries to bedside applications while enhancing the robustness and interpretability of machine learning models.

## 1 Introduction

Neonatal respiratory distress syndrome (NRDS), also known as hyaline membrane disease, is characterized by the formation of a transparent membrane in the lungs. This respiratory condition is caused by a deficiency of pulmonary surfactant (PS) and lung immaturity. NRDS typically presents within 4–6 h after birth and can rapidly worsen within 48–72 h (1). PS is mainly composed of phospholipids, which are synthesized by type II alveolar cells. PS can significantly reduce alveolar surface tension and prevent alveolar collapse at the end of exhalation. PS is synthesized by type II alveolar epithelial cells in the fetal lung from approximately 20–24 weeks of gestation, and pulmonary maturity is generally reached at 35–36 weeks of gestation. Infants with NRDS often have primary or secondary PS deficiency, presenting with shortness of breath (≥60 times/minute), expiratory moaning, nasal flaring, respiratory distress, and cyanosis. In severe cases, apnea and hypotonia may also occur (1, 2). NRDS affects about 7% of full-term infants (3), but it is extremely common in premature infants, making it one of the main causes of global neonatal morbidity and mortality (4). The incidence of NRDS increases with decreasing gestational age and is as high as 92% in infants born at 24–25 weeks (5). NRDS can cause short-term complications, such as pulmonary hemorrhage and intracranial hemorrhage (6, 7), as well as long-term sequelae, such as bronchopulmonary dysplasia and cerebral palsy (7–9), which placing a heavy burden on families and society. Effective clinical management of NRDS relies on prompt diagnosis and intervention. However, the current diagnostic methods, largely reliant on clinical scoring systems and imaging techniques, frequently exhibit limitations in terms of early sensitivity and accuracy in prognostic stratification. This diagnostic challenge underscores the pressing need for objective, molecular-level diagnostic tools. In light of this backdrop, biomarkers have emerged as a significant area of research, potentially enhancing early risk assessment and enabling more individualized management of NRDS (10). ABCA3 mutations are linked to altered surfactant composition and function, and cyclosporin A is a potential ABCA3-targeted molecular corrector (11, 12). In addition, oxidative stress markers provide a basis for grading disease severity (13). These advancements could facilitate the future creation of more accurate diagnostic and management strategies for NRDS, although the existing evidence is still in its early stages. This article reviews the advancements in biomarker research related to NRDS, focusing on inflammatory cytokines, microRNAs, proteins and their clinical applications. The objective of this review is to summarize the existing evidence and identify areas that necessitate further validation prior to broader clinical application.

## 2 Literature search strategy

We conducted a systematic literature search for studies published between January 2010 and December 2025 in PubMed, Web of Science, and Embase. The search combined Medical Subject Headings (MeSH) terms and keywords related to “neonatal respiratory distress syndrome”, “biomarkers”, and “preterm infants”. Specific terms included: (“Respiratory Distress Syndrome, Neonatal” [MeSH] OR “NRDS” OR “hyaline membrane disease”’) AND (“biomarkers” [MeSH] OR “biomarker” OR “cytokine” OR “microRNA” OR “miRNA” OR “protein” OR “peptide” OR “lung ultrasound score”) AND (“infant, premature” [MeSH] OR “preterm infant” OR “preterm”).

Inclusion criteria were: (1) original research articles or peer-reviewed reviews; (2) studies on the diagnostic, prognostic, or therapeutic potential of biomarkers in NRDS; (3) studies involving human subjects (primarily preterm infants) or relevant NRDS animal models; and (4) articles published in English. Exclusion criteria included case reports, conference abstracts, editorials, and studies that did not perform explicit subgroup analyses for neonatal respiratory distress syndrome (NRDS) or focused on other forms of neonatal respiratory disease (e.g., transient tachypnoea of the newborn).

The reference lists of included articles were manually screened for additional relevant studies. The initial search was conducted by two independent reviewers, resolving discrepancies through discussion. Biomarkers were included based on their relevance to diagnosis, severity assessment, prognosis, treatment response, or therapeutic implications in NRDS. This search strategy provided the foundation for the subsequent review of inflammation-related cytokines, microRNAs, protein and peptide biomarkers, and auxiliary diagnostic tools presented in this paper.

## 3 Primary biomarker categories and research advances

**Figure 1:**
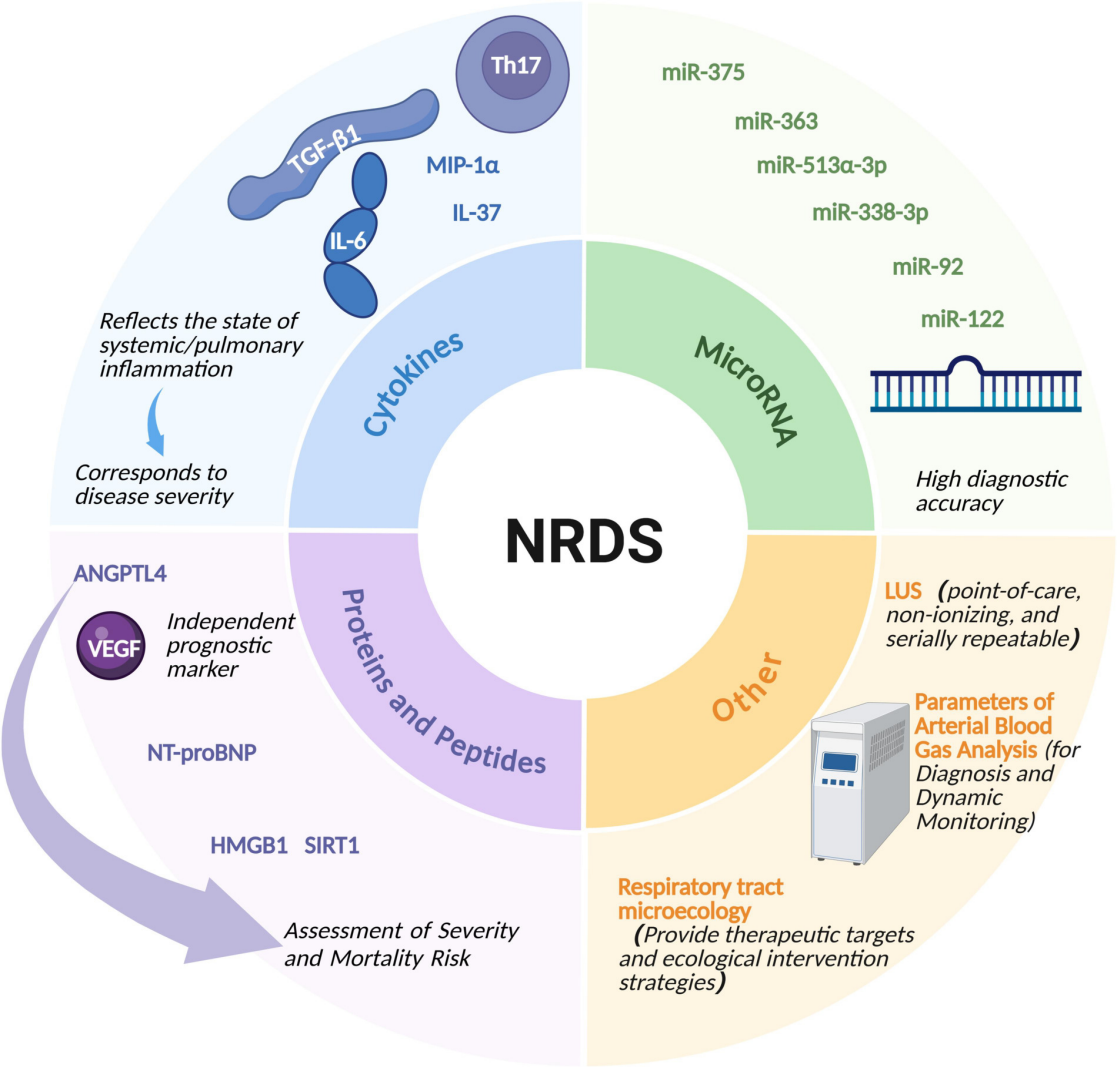
Classification of biomarkers for NRDS and their clinical correlations.

NRDS is a leading cause of morbidity and mortality in preterm infants, primarily characterized by PS deficiency and subsequent inflammatory lung injury. Recent advances in molecular biology and omics technologies have expanded NRDS research beyond traditional clinical and imaging diagnostics, enabling the exploration and application of multi-level biomarkers. These biomarkers may clarify the molecular mechanisms underlying disease onset and progression and provide tools for diagnosis, severity stratification, and prognostic evaluation. For clarity, the biomarkers reviewed below are categorized by their predominant clinical relevance, acknowledging that some may have overlapping diagnostic, severity-related, and prognostic roles. Generally, biomarkers valuable for severity stratification, treatment response assessment, or outcome prediction are of greater practical relevance in NRDS, which is often initially identified based on clinical findings and imaging. This chapter reviews the major categories of biomarkers for NRDS and their research progress, focusing on inflammation-related cytokines, microRNAs, protein and peptide biomarkers, as well as other auxiliary biomarkers, such as lung ultrasound score (LUS), blood gas parameters, and the respiratory microbiome. The discussion summarizes recent developments in this field and highlights their potential translational relevance (Figure 1). To facilitate comparative synthesis of the available evidence, the main biomarkers discussed in this review are summarized in Table 1 according to study population, sample size, reported diagnostic performance, and validation status. The studies reviewed in this review exhibit significant variability in design, study population, and validation levels. As summarized in Table 1, the existing evidence encompasses clinical cohort studies, animal models, omics-based exploratory studies, imaging meta- analyses, and prediction model research. Additionally, sample sizes vary considerably among studies and remain limited for several candidate biomarkers. Most molecular biomarkers discussed lack independent or external validation, whereas lung ultrasound is supported by a more extensive clinical evidence base and systematic synthesis.

**Table 1:** Summary of biomarkers investigated in neonatal respiratory distress syndrome: study population, sample size, diagnostic performance, and validation status.

Biomarker	Population/Model	Sample size	AUC	Validation status	Classification
Th17	NRDS infants vs. healthy newborns; analysis of severe NRDS vs. non-NRDS	82 NRDS + 82 controls	0.8690 (for distinguishing NRDS from non-NRDS, 95% CI 0.8125–0.9255); 0.9192 (for distinguishing severe NRDS from non-NRDS, 95% CI 0.8582–0.9802)	No independent or external validation reported	Exploratory biomarker
TGF-β1	NRDS infants (PS group: 45; non-PS group: 30) + preterm infants without NRDS as controls	75 NRDS + 32 controls	Not reported; mainly reports dynamic changes and correlation with disease severity	No independent or external validation reported	Exploratory biomarker
IL-6	NRDS infants (PS group: 45; non-PS group: 30) + preterm infants without NRDS as controls	75 NRDS + 32 controls	Not reported; mainly reports dynamic changes and correlation with disease severity	No independent or external validation reported	Exploratory biomarker
IL-37	LPS-induced neonatal ARDS mouse model	30 neonatal mice	Not applicable; this is an animal study and no ROC/AUC analysis was performed	No clinical validation	Preclinical candidate biomarker
MIP-1α	Neonatal acute respiratory distress syndrome (NRDS) infants vs. healthy controls	96 NRDS + 60 controls	0.949 (for diagnosing NRDS from controls)	No independent or external validation reported	Exploratory biomarker
miR-375	Umbilical cord blood of NRDS vs. non-NRDS neonates	90 NRDS + 90 controls	0.917 (for distinguishing NRDS from non-NRDS)	No independent or external validation reported	Exploratory biomarker
miR-363	Preterm neonates with NRDS vs. healthy neonates	104 NRDS + 76 controls	0.984 (for the combined model of miR-363 + arterial blood gas parameters + LUS score in diagnosis); 0.936 (for the combined model in prognostic prediction). The exact single-marker AUC of miR-363 was not clearly reported in the original article.	No independent or external validation reported	Exploratory biomarker
miR-513a-3p	NRDS neonates (good vs. poor prognosis)	106 NRDS	0.905 (diagnosis), 0.936 (combined model with blood gas and LUS)	No independent or external validation reported	Exploratory biomarker
miR-338-3p	Neonatal ARDS patients vs. healthy infants	37 ARDS + 32 controls	0.886 (for diagnosing neonatal ARDS)	No independent validation cohort or external validation was reported.	Exploratory biomarker
miR-92 + miR-122	Neonatal ARDS patients, with prognosis assessed as survival vs. death	108 neonatal ARDS patients (77 survivors + 31 deaths)	0.794 for miR-92 alone; 0.840 for miR-122 alone; 0.783 for LUS score alone; 0.920 for miR-92 + miR-122 + LUS score in predicting death (95% CI 0.860–0.977).	No independent validation cohort or external validation was reported.	Exploratory biomarker
ANGPTL4	Preterm infants with NRDS vs. non-NRDS preterm infants; neonates with NRDS for severity and prognosis assessment	128 NRDS + 128 non-NRDS PIs in the diagnostic/prognostic ANGPTL4 study; 115 NRDS + 30 controls in the ANGPTL4 + LUS study	0.902 for diagnosis of NRDS; 0.741 for diagnosis of severe NRDS; 0.854 for predicting death in NRDS infants; 0.938 for ANGPTL4 + LUS score in predicting NRDS prognosis. The combined model was also reported to have higher value for severity assessment than either test alone, but the exact combined AUC for severity was not clearly reported in the original article.	No independent validation cohort or external validation was reported.	Exploratory biomarker
NT-proBNP + HMGB1 + SIRT1	Infants with NRDS vs. normal newborns; prognosis assessed as excellent vs. poor	80 NRDS + 80 controls	0.903 for NT-proBNP alone; 0.829 for HMGB1 alone; 0.794 for SIRT1 alone; 0.958 for the combined model in diagnosis of NRDS; 0.810 for NT-proBNP alone, 0.813 for HMGB1 alone, 0.741 for SIRT1 alone, and 0.935 for the combined model in predicting poor prognosis.	No independent validation cohort or external validation was reported.	Exploratory biomarker
VEGF	Preterm neonates with NRDS vs. preterm neonates without NRDS	80 NRDS + 70 controls	0.949 for distinguishing neonates with RDS from those without RDS; sensitivity 87.50%, specificity 87.14%	No independent validation cohort or external validation was reported.	Exploratory biomarker
Differentially expressed peptide segments (peptidomics)	Umbilical cord blood from preterm newborns with NRDS vs. controls	3 NRDS + 3 controls	Not reported in the original article. The study identified 251 differentially expressed peptides, including 139 upregulated and 112 downregulated peptides, and reported that 11 stable differentially expressed peptides might serve as preclinical biomarkers.	No independent validation cohort or external validation was reported.	Preclinical candidate biomarkers
LUS score	Meta-analysis of studies evaluating lung ultrasound for NRDS diagnosis	9 studies; 703 infants	Pooled sensitivity 0.99, pooled specificity 0.95, and SROC AUC 0.99 for NRDS diagnosis.	This was a systematic review and meta-analysis rather than an external validation study.	Evidence-supported imaging tool
Umbilical cord arterial pH	Full-term newborns with reassuring Apgar score >7 at 5 min, evaluated for respiratory distress syndrome	352 full-term newborns	ROC analysis identified a cut-off of 7.12 for predicting respiratory distress syndrome, with sensitivity 68% and specificity 63%. The original article did not clearly report the AUC value.	No independent validation cohort or external validation was reported.	Exploratory biomarker
Respiratory tract microbiome (16S rRNA sequencing)	Preterm infants with NRDS; pharyngeal swabs and lower respiratory tract sputum collected at 1–3 days and 7–10 days after admission	18 preterm infants with NRDS	Not reported in the original article. The study described microbial composition, richness, diversity, and differential taxa, but did not report ROC/AUC analysis.	No independent validation cohort or external validation was reported.	Exploratory microbiome marker
LUS + clinical indicators (OI, RI, SOFA) machine-learning model	Neonates admitted to the NICU, including NRDS and non-NRDS neonates; model developed for NRDS severity prediction	230 neonates in the derivation cohort, including 119 NRDS and 111 non-NRDS; an independent external validation set included 44 NRDS and 43 non-NRDS neonates.	0.894 for the RF model; 0.841 for LR; 0.828 for NN; 0.726 for SVM.	Internal model development was performed, and an independent external validation set was used.	Exploratory prediction model
Lecithin/sphingomyelin (L/S) ratio predicted by ATR–FTIR + machine learning	Technical proof-of-concept model using purified reagents to simulate nRDS biomarker ratios	259 spectral measurements, including 155 spectra in the calibration set and 104 spectra in the test set.	Not reported as ROC/AUC in the original article. The best-performing three-factor PLSR model showed *R*^2^ = 0.967 and MSE = 0.014 for predicting L/S ratios.	No clinical validation cohort or external clinical validation was reported.	Preclinical technical platform
Oxidative stress biomarkers + SNPs of antioxidant enzymes with machine-learning algorithms	Preterm neonates admitted to intensive care; machine-learning models developed to predict RDS and significant alterations in liver functions	The exact number of neonates was not clearly reported in the original article sections retrieved for verification.	0.600 for the Bayesian network model in predicting RDS; 0.630 for the C5.0 model in predicting significant alterations in liver functions.	No independent validation cohort or external validation was reported; the article explicitly stated that validation in prospective studies is needed.	Exploratory prediction model
RDS-related pulmonary hemorrhage risk model	Extremely preterm infants with RDS, with the outcome defined as pulmonary hemorrhage	309 patients with RDS, including 48 with PH and 261 without PH.	0.868 for the RF model; 0.817 for LR; 0.769 for XGBoost.	Internal model development with four-fold cross-validation was reported; no independent external validation cohort was reported.	Exploratory prediction model

### 3.1 Inflammation-related cytokine biomarkers

Inflammation is a significant factor in the development and progression of NRDS. Inflammatory cytokines can harm alveolar epithelial cells and capillary endothelial cells, leading to a decrease in the synthesis or an increase in the inactivation of pulmonary surfactant. Additionally, these cytokines may trigger pulmonary edema and interstitial fibrosis, which can hinder lung ventilation and gas exchange in newborns. Due to the structural and functional immaturity of the lungs in premature infants, they are particularly vulnerable to inflammatory injury, which can exacerbate respiratory distress (14). Inflammation-related biomarkers may provide more insight for assessing severity and prognosis than for diagnosis alone. Several reports have linked inflammatory cytokines like TGF-β1 and IL-6 to disease severity in premature infants with NRDS (15). Furthermore, increased levels of inflammatory mediators such as Th17-related cytokines, TGF-β1, IL-6, IL-37, and MIP-1α indicate the presence of pulmonary inflammation and may correlate with the severity and prognosis of the disease. The majority of existing research in this field consists of observational and single-center studies, and there is a notable absence of independent validation for most of these markers.

#### 3.1.1 Th17

Th17 cells are a subset of helper T cells and participate in a variety of inflammatory disease processes, especially in acute lung injury (16). IL-23 and IL-17 are Th17-related cytokines. IL-23 activates Th17 cells, promoting their proliferation, differentiation, and survival. Activated Th17 cells primarily secrete IL-17, which recruits neutrophils and other inflammatory cells, amplifying the inflammatory response and worsening lung inflammation and tissue damage. Compared to the healthy control group, serum levels of IL-17 and IL-23 were significantly higher in infants with NRDS, suggesting activation of this inflammatory pathway in the disease. The levels of these cytokines were closely associated with disease severity, with concentrations in severe NRDS patients significantly exceeding those in mild disease, and both were positively correlated with disease severity. In terms of diagnostic value, ROC analysis showed that the AUC for IL-17 and IL-23 was 0.9192 (95% CI, 0.8582–0.9802) and 0.8575 (95% CI, 0.8125–0.9255), respectively, for distinguishing infants with NRDS, particularly those with severe disease (17). These findings suggest that IL-17 and IL-23 may be associated with NRDS severity and could serve as early diagnostic biomarkers. However, the available evidence is still limited to single-center studies without independent validation.

#### 3.1.2 TGF-β1 and IL-6

Transforming growth factor-β1 (TGF-β1) is a cytokine with multiple biological activities and is widely involved in regulating cell proliferation and differentiation. It is primarily secreted by macrophages, epithelial cells, endothelial cells, and fibroblasts. Studies have shown that TGF-β1 plays a significant role in the pathogenesis and progression of many diseases, particularly lung diseases, by mediating acute or chronic lung injury through the activation of relevant signaling pathways (18). As a key inflammatory factor, IL-6 is considered a core inflammatory mediator for adult acute respiratory distress syndrome (ARDS) (19). IL-6 induces T cells to differentiate into Th17 cells, thereby inhibiting the differentiation of Treg cells and resulting in an imbalance between Treg cells and Th17 cells (20). Therefore, both TGF-β1 and IL-6 play important roles in the pathophysiology of ARDS. In contrast, the primary cause of NRDS is surfactant deficiency, with inflammatory responses often contributing significantly to this depletion. Feng et al. (15) compared the expression levels of TGF-β1 and IL-6 in the pulmonary tissue of infants with NRDS and healthy newborns. The results indicated that both TGF-β1 and IL-6 were detected in normal lung tissue. On days 1 and 3, the mean expression levels of TGF-β1 and IL-6 in infants with NRDS were significantly elevated compared to those in the control group, and there was a positive correlation between these levels and the severity of NRDS. These findings imply that TGF-β1 and IL-6 may play a role in the severity of the disease and could be valuable for early risk stratification. These findings suggest that TGF-β1 and IL-6 may be associated with disease severity and could be useful for early risk stratification. Currently, the available studies are limited in scale, and independent validation has not been reported.

#### 3.1.3 IL-37

IL-37 is an anti-inflammatory member of the IL-1 family (21). In a neonatal acute respiratory distress syndrome model induced by LPS, IL-37 was associated with a decrease in the expression of inflammatory cytokines, the inhibition of NLRP3 inflammasome activation, and the suppression of the CXCR4/SDF-1 signaling pathway (22). These findings suggest that IL-37 may be involved in the regulation of inflammatory responses relevant to neonatal lung injury. However, the existing evidence primarily comes from experimental models rather than clinical cohorts, and its effectiveness as a biomarker in NRDS has yet to be established.

#### 3.1.4 MIP-1α

Pulmonary surfactant deficiency can compromise the integrity of the alveolar-capillary barrier, trigger localized immune responses, and play a significant role in the development of NRDS. Macrophage inflammatory protein-1α (MIP-1α), a key inflammatory mediator in this process, recruits monocytes and T cells to the lungs through binding to CCR1/CCR5 receptors and thereby amplifies the local inflammatory response (23). Inflammatory infiltration damages the alveolar structure and induces pulmonary edema, activates NF-κB and ERK1/2 signaling pathways, amplifies oxidative stress and inflammatory mediator release, creates a positive feedback loop, and worsens lung injury (24). The study by Li et al. highlighted the clinical significance of MIP-1α. Their findings revealed that infants diagnosed with NRDS exhibited markedly elevated levels of MIP-1α in their umbilical cord blood compared to the healthy control group. Furthermore, the concentration of MIP-1α was positively correlated with both the severity of the disease and the prognosis (25). Therefore, MIP-1α may have potential diagnostic and prognostic value in NRDS. However, the level of this molecule may be influenced by common interventions such as mechanical ventilation and oxygen therapy, which limits its specificity as an independent diagnostic indicator (24, 25). Future clinical applications may necessitate incorporating it into a multifactorial model to minimize potential interference and improve diagnostic accuracy. While MIP-1α appears to play a significant role in the inflammatory cascade associated with NRDS, its development into a dependable clinical tool requires additional validation in prospective cohorts, accounting for relevant confounding factors.

### 3.2 MicroRNA biomarkers

MicroRNAs (miRNAs) are endogenous non-coding single-stranded RNA molecules that regulate gene expression post-transcriptionally by targeting specific mRNAs. They play key roles in physiological and pathological processes such as cell differentiation, proliferation, cell cycle regulation, and organ development (26). Their expression is specific to tissues, and abnormal expression is often linked to various disease states. In NRDS research, miRNAs have gained attention for their role in disease pathogenesis and their potential as biomarkers in clinical assessment. Some miRNAs may be especially useful for identifying disease severity and predicting short-term prognosis when considered alongside other clinical indicators. For example, miR-338-3p has shown potential relevance to neonatal lung injury in ARDS-related studies (27), whereas miR-375 has been reported to be closely associated with disease severity in NRDS and may serve as an auxiliary biomarker (28). These findings suggest that specific miRNAs may be valuable for predicting, diagnosing, and assessing the prognostic of NRDS. However, most available studies are single-center and based on relatively small cohorts, with independent validation lacking in many reports. Additionally, some of the existing evidence is derived from neonatal ARDS rather than classical surfactant-deficient NRDS.

#### 3.2.1 miR-375

MiR-375 regulates pulmonary surfactant depletion by affecting its secretion from type II alveolar epithelial cells through cytoskeletal remodeling and inhibiting the Wnt/β-catenin pathway, disrupting normal transdifferentiation of these cells (29, 30). In clinical practice, Deng et al. found that miR-375 expression was significantly upregulated in the umbilical cord blood of infants with NRDS. This expression was positively correlated with disease severity, showing significantly higher levels in severely affected infants compared to those with milder forms of the condition (28). This study further showed that miR-375 had high diagnostic efficacy in distinguishing between NRDS and non-NRDS newborns (AUC = 0.917), and its elevated expression was closely related to adverse clinical outcomes such as ventilator dependence and persistent respiratory distress. These findings suggest that miR-375 may be involved in NRDS pathogenesis and could indicate disease severity and prognosis. However, the available evidence is limited to single-center data, and its role as a clinically useful biomarker requires independent validation.

#### 3.2.2 miR-363 and miR-513a-3p

In diagnosing and assessing the prognosis of NRDS, miR-363 and miR-513a-3p have shown promise as biomarkers. Hou et al. found that miR-363 expression in infants with NRDS was significantly lower than in healthy newborns, with an AUC of 0.879, sensitivity of 71.2%, and specificity of 86.8%, indicating potential diagnostic value as a single marker in that cohort (31). In contrast, miR-513a-3p expression was increased in infants with NRDS, particularly in those with poor prognosis, showing an AUC of 0.905, a sensitivity of 76.9%, and a specificity of 88.9%. This suggests that this marker may be relevant for assessing disease severity and prognosis (32). The expression levels of these two miRNAs were significantly correlated with arterial blood gas parameters such as pH, PaO_2_, and PaCO_2_. In two single-center studies, combining these markers with LUS and blood gas analysis showed higher diagnostic and prognostic performance than relying on a single indicator. The combined miR-363 model achieved an AUC of 0.984, with a sensitivity of 94.2% and a specificity of 93.4% (31); whereas the combined miR-513a-3p model yielded an AUC of 0.936, with a sensitivity of 85% and a specificity of 89% (32). These findings suggest that combined approaches could be valuable for assessing severity and prognosis. However, the existing evidence is still preliminary and necessitates independent validation.

#### 3.2.3 miR-338-3p, miR-92, and miR-122

In studies of neonatal ARDS, miR-338-3p, miR-92 and miR-122 have shown potential biological relevance (27, 33), especially in inflammatory responses, immune regulation, and apoptosis. Studies show that miR-338-3p expression is significantly reduced in neonatal ARDS and is closely associated with disease severity, clinical indicators like the PaO_2_/FiO_2_ ratio and oxygenation index, and inflammatory cytokines such as IL-1β, IL-6, and TNF-α. In addition, miR-338-3p may be a potential therapeutic target in neonatal ARDS by inhibiting LPS-induced alveolar epithelial cell injury and reducing apoptosis (27). Unlike miR-338-3p, miR-92 and miR-122 may be involved in neonatal ARDS through different mechanisms. miR-92 has been reported to regulate lung inflammation through the PTEN/AKT/NF-κB signaling pathway (34); whereas miR-122 may contribute to neonatal ARDS progression by promoting inflammation and apoptosis (33). Higher expression levels of miR-92 and miR-122 have been linked to poorer prognosis and an increased risk of death in neonatal ARDS. Therefore, regulating miR-92 and miR-122 may offer a potential therapeutic strategy for neonatal ARDS. Although existing studies have investigated the roles of these miRNAs in neonatal ARDS, specific evidence related to NRDS remains limited. Given the significant differences in pathophysiology between classical surfactant-deficient NRDS and neonatal ARDS—particularly regarding surfactant deficiency, lung development, and inflammatory injury—future studies should further explore the functional roles of miR-338-3p, miR-92, and miR-122 in well-defined NRDS cohorts and clarify the underlying mechanisms. In addition, the combined detection of these miRNAs may be valuable in neonatal ARDS; however, their diagnostic potential in NRDS has yet to be established and should not be directly inferred from evidence based on neonatal ARDS.

### 3.3 Protein and peptide biomarkers

Protein and peptide biomarkers have become an important area of research in NRDS. Studies on specific markers, such as angiopoietin-like protein 4 (ANGPTL4) and vascular endothelial growth factor (VEGF), suggest potential value for diagnosing, prognostic evaluation, and treatment response assessment in NRDS. As factors closely related to pulmonary development and injury, ANGPTL4 and VEGF are associated with disease severity and survival outcomes in neonates. Clinically, these biomarkers may be particularly relevant for severity stratification and outcome assessment. However, most available studies are still single-center and lack independent validation. In addition, some studies have proposed novel biomarkers for NRDS by analyzing peptide profiles in neonatal umbilical cord blood. These peptides may reflect pathological processes such as abnormal lung development and respiratory failure, providing insight into relevant mechanisms through functions related to extracellular matrix regulation and cell adhesion. However, the peptide evidence is still in early stages and based on very limited sample sizes.

#### 3.3.1 Angiopoietin-like protein 4 (ANGPTL4)

ANGPTL4 is a member of the angiopoietin-like protein family, which regulates lipid and glucose metabolism. It also plays a key role in inflammatory responses and vascular permeability (35, 36). In NRDS, ANGPTL4 expression is significantly increased and closely related to disease severity. Studies show that elevated ANGPTL4 expression promotes lung inflammation and vascular permeability, resembling the pathological features of acute lung injury, including ARDS (37, 38). Although the exact mechanism of ANGPTL4 in NRDS is not fully clarified, its role in lung inflammation and vascular permeability suggests it may act through similar pathways in NRDS. Serum ANGPTL4 levels are associated with the severity and prognosis of NRDS. The AUC for the diagnosis of NRDS was 0.902 (sensitivity, 88.28%; specificity, 86.72%), whereas the AUC for the diagnosis of severe NRDS was 0.741 (sensitivity, 66.67%; specificity, 79.52%) (39). In addition, the mortality rate in the high-expression group was significantly higher than that in the low-expression group (61.8% vs. 6.8%) (39). When ANGPTL4 was combined with LUS, the prognostic AUC increased to 0.938 (sensitivity, 91.7%; specificity, 91.1%) (40). These findings imply a potential association between ANGPTL4 and the severity and prognosis of NRDS. Nevertheless, the existing evidence is primarily derived from single-center studies, and further validation in larger cohorts is necessary to establish its clinical relevance.

#### 3.3.2 NT-proBNP, HMGB1, SIRT1

N-terminal pro-brain natriuretic peptide (NT-proBNP) is a peptide biomarker secreted by myocardial cells, primarily reflecting the increase in cardiac load resulting from compromised lung function. It may aid in the assessment of pulmonary circulatory pressure and cardiac dysfunction (41). High mobility group box 1 (HMGB1) is an inflammatory mediator involved in the pulmonary inflammatory response in NRDS, and elevated HMGB1 levels are associated with disease severity and prognosis (42). Sirtuin 1 (SIRT1) is a deacetylase that regulates key processes such as autophagy and cell repair and may play an important role in lung injury repair during NRDS (41). These three markers reflect various aspects of NRDS pathophysiology, including cardiac function, inflammation, and cellular repair. Combined assessment of NT-proBNP, HMGB1, and SIRT1 may provide complementary information on NRDS severity and may improve diagnostic performance in the reported study (42). Currently, this evidence is limited and has not yet undergone independent validation.

#### 3.3.3 VEGF

Vascular endothelial growth factor (VEGF) is a protein cytokine that plays a crucial role in promoting angiogenesis. It serves as a key regulatory factor in lung maturation and is extensively involved in early lung development and angiogenesis. Research indicates that VEGF levels in infants with NRDS are significantly lower than those in a healthy control group, with infants suffering from severe disease exhibiting even lower VEGF levels compared to those with mild disease. ROC curve analysis showed that VEGF has a sensitivity of 87.5% and a specificity of 87.14% in differentiating infants with NRDS from healthy newborns, suggesting its potential as a candidate biomarker for NRDS (43). In addition, VEGF levels were associated with neonatal prognosis. Lower VEGF levels were associated with poorer outcomes in the reported study (43). This finding still requires confirmation in independent cohorts.

#### 3.3.4 Differentially expressed peptide segments

Peptidomic analysis is being increasingly utilized in neonatal disease research, particularly in the context of early diagnosis and the investigation of underlying mechanisms. For instance, in the study of hypoxic-ischemic encephalopathy, prior research has identified pertinent endogenous peptides through this approach (44). In the field of NRDS, a study based on liquid chromatography-tandem mass spectrometry of umbilical cord blood identified 251 differentially expressed peptides, including 139 upregulated peptides and 112 downregulated peptides (fold change ≥ 2.0, *P* < 0.05) (45). Gene Ontology (GO) analysis showed that these peptides were primarily involved in biological processes related to NRDS pathology, such as extracellular matrix organization, cell adhesion, and proliferation. Kyoto Encyclopedia of Genes and Genomes (KEGG) pathway analysis indicated connections to NRDS-related pathways, including the PI3K-Akt signaling pathway. The study also identified 11 stably upregulated peptides with a long half-life and high stability, functionally associated with lung injury, cell adhesion, and oxidative stress, suggesting these peptides may serve as promising biomarker candidates for NRDS (45). However, these findings are derived from a limited discovery-stage study and have not yet undergone independent validation.

### 3.4 Additional auxiliary biomarkers

**Figure 2:**
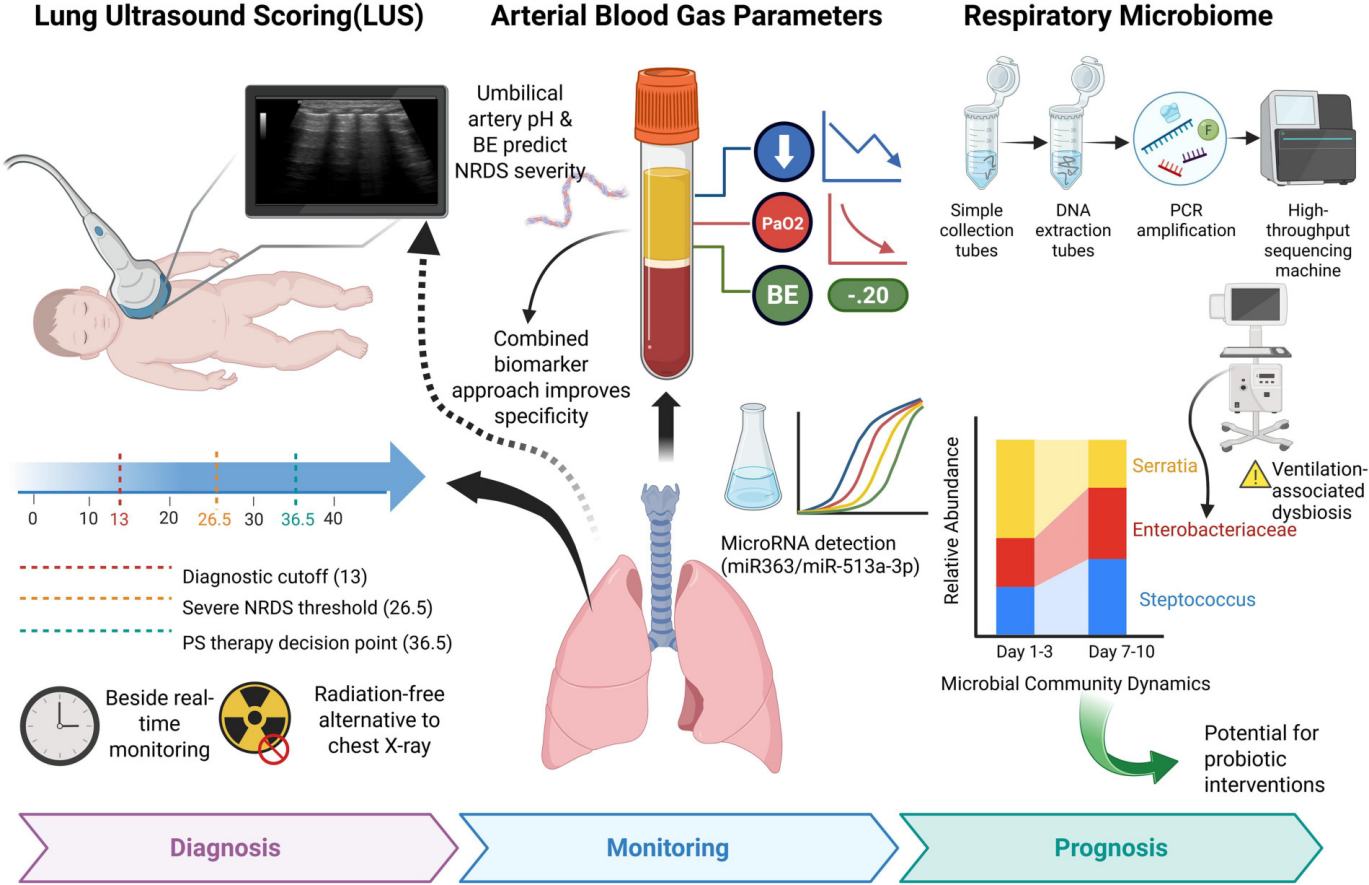
Auxiliary assessment tools for NRDS: comprehensive application of lung ultrasound score, arterial blood gas analysis, and respiratory microbiome.

In addition to the aforementioned biomarker categories, several auxiliary indicators have demonstrated potential clinical relevance in the diagnosis and prognostic evaluation of NRDS. Given that NRDS is frequently identified based on clinical presentation and imaging findings, these auxiliary markers could be particularly valuable when integrated with bedside assessment tools for evaluating severity, assessing treatment responses, and making prognostic judgments. In recent years, LUS, as a non-invasive, radiation-free, and rapid bedside assessment tool, has gained prominence in clinical practice and may facilitate real-time evaluation of disease severity (46). Arterial blood gas analysis provides an important clinical basis by dynamically monitoring core indicators such as pH, PaO_2_, and PaCO_2_ (31). Changes in the respiratory microbiota, especially those influenced by interventions such as mechanical ventilation, also provide a new perspective for understanding and managing NRDS (47). These biomarkers and tools may offer complementary information for assessing severity, evaluating prognosis, and managing NRDS on an individualized basis. However, the available evidence is not consistent across the various categories. Lung ultrasound has been evaluated in larger clinical studies and meta-analyses, while arterial blood gas parameters provide supportive rather than disease-specific insights. Additionally, microbiome-related findings are currently derived from small exploratory studies (Figure 2).

#### 3.4.1 Lung ultrasound score (LUS)

In clinical practice, the diagnosis of NRDS usually relies on a thorough assessment of clinical manifestations, imaging findings, and arterial blood gas analysis. However, newborns are in a critical phase of rapid growth and development, making them particularly susceptible to ionizing radiation and associated biological injury. This sensitivity restricts the repeated use of chest x-rays for ongoing monitoring in the diagnosis and management of NRDS (48). LUS offers significant advantages in diagnosing NRDS due to its ease of use, non-invasive nature, lack of radiation exposure, and suitability for real-time bedside evaluation. It has become an increasingly important diagnostic tool in clinical practice. Hua et al. (49) found that, when LUS was used to distinguish non-NRDS from NRDS in neonates, an optimal cutoff value of 13 yielded an AUC of 0.938. When distinguishing non-severe from severe NRDS, the optimal cutoff value was 26.5. In another study, Jiang et al. (50) developed a 14-segment lung scoring system to assess the efficacy of PS therapy in neonatal NRDS. They found that the optimal threshold for PS treatment decision-making was 36.5, with an AUC of 0.99. A meta-analysis (46) further suggested that LUS may provide superior diagnostic performance for NRDS when compared to chest x-ray examinations. Additionally, LUS presents the benefits of being free from radiation and easily repeatable. These findings underscore its potential as an effective bedside imaging tool. However, the utility of LUS may be influenced by factors such as operator dependency and interobserver variability. In contrast, the evidence supporting biomarker-based combination strategies is currently less robust than that for lung ultrasound.

#### 3.4.2 Arterial blood gas analysis parameters

Arterial blood gas analysis is a reliable tool for evaluating neonatal ischemia and hypoxia. Its core parameters (pH, PaO_2_, PaCO_2_, HCO_3_− and BE) dynamically reflect lung ventilation, gas exchange function, tissue oxygenation and metabolic status (31). De et al. (51) found that the reduction of pH and BE in neonatal umbilical artery blood can predict NRDS. However, arterial blood gas analysis is not effective in differentiating NRDS from other neonatal respiratory diseases (such as neonatal pneumonia or wet lung) (31). Therefore, the researchers combined arterial blood gas analysis with LUS to overcome this limitation. Research has shown that combining arterial blood gas analysis with LUS and the detection of miR-363 or miR-513a-3p is associated with greater accuracy in diagnosing NRDS and predicting prognosis in the reported cohorts (31, 32). These findings indicate that the integration of biomarkers with bedside clinical assessment tools may prove beneficial in this setting. However, the current evidence is largely confined to single-center studies and necessitates independent validation.

#### 3.4.3 Respiratory microbiome

Research on NRDS has increasingly focused on changes in the respiratory microbiota. Infants with NRDS typically receive interventions such as pulmonary surfactant replacement therapy, tracheal intubation, and mechanical ventilation. These treatments may alter the respiratory microbial environment, impair airway barrier function, and promote pathogen colonization, contributing to microbial imbalance during NRDS. Studies using 16S rRNA gene sequencing have shown that the composition of the respiratory microbiota remains relatively stable at 1–3 days and 7–10 days after birth in infants with NRDS. The dominant genera include Streptococcus, Enterobacteriaceae, Hafnia, Sarella, and Gasmonas. Moreover, the abundance of Streptococcus and Enterobacteriaceae significantly increased in the later stages of the disease (47).

Changes in the respiratory microbiota may be linked to the clinical manifestations of NRDS. During interventions like mechanical ventilation, microbial balance can be disrupted, promoting the colonization of hospital-associated pathogens, including Gram-negative bacteria (47). Restoring the respiratory microbial balance may present a promising avenue for research in NRDS; however, this remains a hypothesis-generating at this stage, and its therapeutic relevance has yet to be established. Future studies are necessary to further investigate the specific role of the microbiome in NRDS and to assess whether microbiome-based interventions can enhance clinical outcomes. These findings may enhance our understanding of NRDS pathophysiology and help identify potential exploratory biomarker candidates. However, their value for early diagnosis and individualized treatment still needs validation in larger clinical studies.

## 4 Innovative research methods and advances

**Figure 3:**
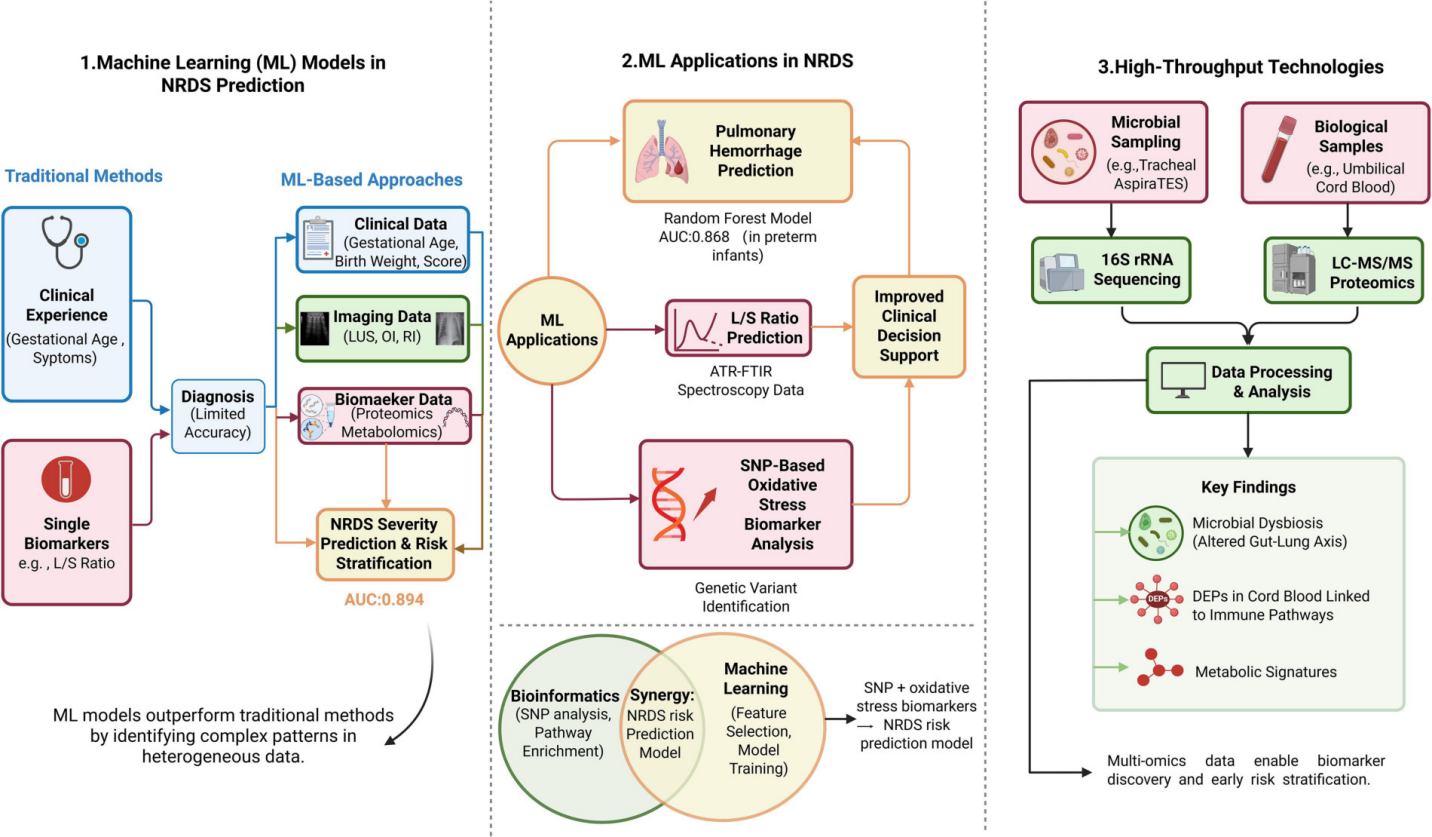
From multidimensional data integration to precision intervention: innovative research approaches for NRDS.

Scientific and technological advancements have significantly propelled NRDS research forward, especially through the application of machine learning algorithms and high-throughput technologies. Traditional prediction methods typically depend on individual clinical indicators, which can restrict their effectiveness. In contrast, machine learning has the capability to synthesize multidimensional data, enabling the identification of intricate disease patterns. This approach not only enhances predictive performance but also facilitates more personalized management strategies (10). High-throughput technologies like mass spectrometry and genomics may help identify biomarkers associated with NRDS and accelerate early diagnosis and mechanistic research (47). These technological innovations may offer new avenues for biomarker discovery and risk assessment in NRDS (Figure 3).

### 4.1 Application of predictive models

Predictive models, especially those based on machine learning algorithms, have shown potential advantages over traditional methods in the diagnosis and severity prediction of NRDS in some studies (10). Traditional methods typically depend on clinical experience and single indicators. In contrast, machine learning has the capacity to integrate clinical data, imaging findings, and biomarkers to identify complex patterns, thereby potentially enhancing predictive performance. One study incorporated clinical data, including LUS, oxygen index (OI), and respiratory index (RI), alongside machine learning algorithms, such as random forest (RF) and support vector machine (SVM), to improve the accuracy of NRDS severity prediction. The AUC of the random forest model achieved a score of 0.894 (52). Ahmed et al. (53) combined machine learning with attenuated total reflectance Fourier transform infrared spectroscopy (ATR-FTIR) to support early diagnosis of NRDS by predicting the alveolar/airway ratio (L/S ratio). Sridharan et al. (13) investigated the use of antioxidant enzyme polymorphisms and oxidative stress biomarkers as predictors for assessing the effectiveness of machine learning algorithms in predicting NRDS and its impact on liver function. Moreover, several studies have implemented predictive models aimed at enhancing the diagnostic evaluation of NRDS-related complications. For example, Liu et al. (54) reported that the random forest (RF) model showed the highest diagnostic performance for predicting pulmonary hemorrhage in extremely preterm infants with NRDS, achieving an AUC of 0.868.

Machine learning models offer advantages over traditional methods by efficiently processing multidimensional data and recognizing complex patterns. They have the potential to enhance early risk prediction for NRDS and its complications in selected settings; however, their generalizability and clinical utility require further external validation.

Several methodological issues must be addressed before these models can be effectively implemented in neonatal intensive care unit (NICU). One significant concern is overfitting, which limits generalizability. Most available studies rely on single-center data with relatively small sample sizes, increasing the risk that a model performs well in the training set but shows reduced performance when applied to new populations. External validation is crucial for assessing model generalizability, yet most NRDS prediction models lack independent external validation, leaving their performance in real-world clinical settings uncertain.

Another issue is model interpretability. More complex machine learning approaches, such as ensemble models and neural networks, can be difficult to interpret clinically, making it challenging to identify which features primarily influence a given prediction. In the context of neonatal critical care, this may hinder clinical confidence and adoption. Future studies should incorporate interpretability methods like SHAP or LIME to better characterize the contributions of individual variables to model outputs. Additionally, greater attention should be given to clinical calibration. An effective prediction model should not only differentiate between high-risk and low-risk patients but also provide risk estimates that align closely with observed outcomes. Currently, many studies prioritize discrimination metrics like AUC, while calibration is assessed less frequently, potentially limiting clinical applicability.

### 4.2 Application of high-throughput technology

High-throughput technology has significantly advanced biomarker research and early diagnosis in NRDS. For example, 16S ribosomal RNA (16S rRNA) gene sequencing of respiratory specimens from infants with NRDS has suggested that early changes in the microbial community may be involved in disease pathogenesis and clinical manifestations (13). At the proteomic level, liquid chromatography-tandem mass spectrometry (LC-MS/MS) has emerged as a crucial technique for identifying and quantifying of biomarkers associated with NRDS. Research indicates that variations in peptide expression found in umbilical cord blood are significantly linked to the progression of NRDS and are implicated in critical biological processes, including immune response and lung development (45).

With the ongoing surge in high-throughput data, a significant challenge in contemporary research is the effective identification of disease-related information within high-dimensional and multisource datasets. The integration of bioinformatics analysis with machine learning models provides a viable solution to address this issue. For example, several studies have merged single-nucleotide polymorphisms (SNPs) in antioxidant enzyme genes with oxidative stress markers to develop predictive models using machine learning, aimed at evaluating the risk of NRDS (13).

The strategic use of high-throughput technologies, such as mass spectrometry, machine learning, and multimodal data integration, has significantly enhanced the identification of NRDS biomarkers and has bolstered early diagnostic capabilities. As these methodologies continue to evolve and become more integrated, they are expected to play a more central role in refining risk stratification and tailoring individualized intervention strategies for NRDS.

## 5 Conclusion

NRDS is a respiratory failure resulting from PS deficiency and lung immaturity, typically appearing within 4–6 h after birth. The disease is more common in premature infants, with incidence increasing as gestational age decreases. It may also lead to complications such as bronchopulmonary dysplasia. Therefore, early diagnosis and prognostic evaluation are crucial for improving clinical outcomes. This article reviews recent advancements in research on NRDS-related biomarkers, focusing on categories such as inflammatory cytokines, miRNAs, proteins, and peptides. Beyond their potential diagnostic value, several of these markers may be more clinically useful for severity stratification, prognostic evaluation, and treatment response assessment when interpreted alongside bedside tools and imaging findings.

In terms of inflammatory cytokines, elevated serum levels of IL-17 and IL-23 have been reported in infants with NRDS and may be associated with disease severity. TGF-β1 and IL-6 have also been reported to be highly expressed in the early stages of the disease and may help identify patients at higher risk. IL-37 possesses anti-inflammatory properties and may have potential relevance in NRDS research, although its diagnostic and therapeutic value remains preliminary. In the realm of miRNAs, miR-375 has been reported to be elevated in the umbilical cord blood of infants with NRDS and may be associated with poorer prognosis. Conversely, miR-363 expression has been reported to decrease, while miR-513a-3p expression increases. When combined with LUS and blood gas analysis, diagnostic accuracy may be further improved. Among protein biomarkers, ANGPTL4 has shown potential diagnostic value for NRDS in preliminary studies and may also be related to disease severity and prognosis. Low VEGF levels may indicate impaired pulmonary vascular development and have been associated with poor prognosis.

In the study, presented, the simultaneous measurement of NT-proBNP, HMGB1, and SIRT1 showed better diagnostic accuracy compared to any single marker. This finding indicates that a multi-marker approach warrants further investigation. Furthermore, LUS, being a non-invasive and radiation-free tool for bedside evaluation, has exhibited promising diagnostic performance in existing research and may serve as a valuable asset for clinical assessments conducted at the bedside. By integrating multidimensional clinical data, machine learning models may provide a novel approach for early prediction and risk stratification in NRDS.

However, several limitations persist in the current literature. Some biomarkers, such as MIP-1α, exhibit limited specificity and can be influenced by factors like mechanical ventilation and oxygen therapy. The available evidence regarding miR-338-3p, miR-92, and miR-122 primarily stems from studies on neonatal ARDS rather than classical surfactant-deficient NRDS. Therefore, caution should be exercised when extrapolating these findings to NRDS, and the specific role of these biomarkers in NRDS remains to be clarified.

The current evidence base presents a diverse range of study designs and populations, with many of the reviewed studies being single-center investigations that involve relatively small sample sizes. Additionally, most molecular biomarkers lack independent or external validation. From a clinical implementation standpoint, the biomarkers discussed in this review are at varying stages of practical application. While LUS and arterial blood gas analysis have already been integrated into routine bedside assessments in the NICU, the majority of molecular biomarkers are not yet suitable for standard use. However, a few candidates, such as ANGPTL4 and certain combined assessment models, are showing promise for clinical translation based on preliminary studies. Nonetheless, even for these markers, independent validation remains absent. For the remaining cytokines, miRNAs, proteins, peptides, and microbiome-related markers, the evidence is predominantly exploratory. Furthermore, data concerning assay availability, turnaround time, cost, and comparative performance against existing clinical tools is still limited for many candidate biomarkers. As a result, their potential clinical utility must be interpreted cautiously and validated through further research.

Therefore, future research should focus on multicenter prospective studies that involve larger sample sizes. This approach will help validate the clinical significance of current biomarkers, further explore multi-biomarker models, clarify the underlying mechanisms, and assess the potential contributions of machine learning and high-throughput technologies in enhancing the assessment of NRDS.
